# Who Participates in Running Events? Socio-Demographic Characteristics, Psychosocial Factors and Barriers as Correlates of Non-Participation—A Pilot Study in Belgium

**DOI:** 10.3390/ijerph14111315

**Published:** 2017-10-28

**Authors:** Delfien van Dyck, Greet Cardon, Ilse de Bourdeaudhuij, Lisa de Ridder, Annick Willem

**Affiliations:** 1 Research Foundation Flanders (FWO), Egmontstraat 5, 1000 Brussels, Belgium; 2 Faculty of Medicine and Health Sciences, Department of Movement and Sports Sciences, Ghent University, Watersportlaan 2, 9000 Ghent, Belgium; Greet.cardon@ugent.be (G.C.); Ilse.debourdeaudhuij@ugent.be (I.d.B.); Annick.willem@ugent.be (A.W.); 3 Department of Applied Health Sciences, University College West Flanders (Howest), Sint-Jorisstraat 71, 8000 Bruges, Belgium; Lisa.de.ridder@howest.be

**Keywords:** exercise, socio-ecological framework, adults, physical activity, Belgium

## Abstract

In Western countries, the popularity of running events has increased exponentially during the last three decades. However, little is known about the profile of non-participants. This knowledge is crucial to tailor promotional actions towards people who are currently not participating. Therefore, this study aimed: (1) to examine which socio-ecological factors are related to participation in running events, (2) to give an overview of the barriers towards participation, and (3) to examine differences in barriers depending on gender, age and educational level. Flemish adults (*n* = 308) completed an online questionnaire about their socio-demographic and psychosocial characteristics, physical activity levels, participation in running events and barriers towards participation. Results showed that motivation, family social support, knowledge about running events and physical activity levels were associated with participation in running events. Among non-participants, the main barriers were bad physical condition, lack of time and lack of interest. In participants, lack of time, distance to the event and financial barriers were most prevalent. With some exceptions, barriers were relatively similar across socio-demographic subgroups. This study confirmed a democratization among participants of running events and provided evidence about which barriers should be tackled to increase participation among population subgroups that are currently underrepresented in such events.

## 1. Introduction

Since the 1960s, the “running sport” has been growing internationally as part of a specific trend defined as “desportification and deinstitutionalization of the sport sector” [1,2,3]. This trend implies that adults no longer engage in sports because of the competitive aspect, but mainly because sports are healthy, relaxing, adventurous or pleasant [1,4,5]. Consequently, running developed into an independent sport, dissociated from organized athletics. In the same line, the popularity of running events (e.g., city runs, park runs, trail runs, obstacle runs) increased exponentially in most Western countries during the last three decades. For instance, the number of finishers in the 20 largest road races worldwide doubled from 866,000 in 2001 to almost 1,600,000 in 2012 [5]. Running events are also very popular in Flanders (the northern part of Belgium; 6.5 million inhabitants; 481.4 inhabitants/km²): about 600 running events are organized annually, with a total of more than 250,000 finishers [6]. Remarkably, the number of short runs (up to 5.6 km) has increased considerably in Flanders since the early 1990s, whereas the number of events covering longer distances has remained relatively stable. Currently an almost equal number of “short distance” and “longer distance” runs are being organized in Flanders [6]. 

In health research, there has been an ongoing discussion about the potential of running events to increase overall sport participation and physical activity among adults [7]. Organizers of running events as well as the sport and recreation industry in general, claim there is an exercise-enhancing effect of participating in such events, but empirical evidence supporting such claims is not very consistent [8]. Some studies carefully confirm a potential positive effect on physical activity [7,8,9,10], but long-term effects have rarely been examined [9]. Furthermore, potential positive effects seem to be dependent on socio-demographic factors, motivation and prior physical activity levels [8]. 

Notwithstanding this mixed evidence and unresolved debate, some important advantages of running events should be highlighted. First, running events can certainly act as a stimulus for increased pre-event physical activity: the majority of participants trains in preparation for such events, and although it is not clear whether these increased physical activity levels persist post-event, they may be associated with specific health benefits [8,11]. Second, running events have the capacity to promote interest in active leisure and to increase positive attitudes towards physical activity on a population level [8]. Third, as running events encourage physical activity within a social context they can indirectly increase feelings of social inclusion and have an impact on psychosocial wellbeing [10]. This inherent feeling of social inclusion may be particularly valuable for specific population segments that are known to be less active (e.g., ethnic minorities, older adults, women) [12]. 

Because of these known benefits associated with participation in running events, it is important to continue promoting participation in such events. In order to optimize promotional strategies and tailor them towards population subgroups that are currently underrepresented in running events, it is necessary to gain more detailed insight into the profile of non-participants: who is currently not participating and what prevents non-participants from participation? Previous studies in the domain of running events focused almost exclusively on characteristics of participants [7,8,9,10] while a comparison with non-participants is crucial in order to understand the bigger picture. 

Some evidence is available regarding the socio-demographic profile of participants, compared with non-participants. Between 1969 and now there has been a “democratization” among participants of running events [6]. The mean age has increased and currently, the majority of runners in Flanders correspond to the 30–50 year old group. Furthermore, although men are still overrepresented (e.g., 57% versus 43% women in Flanders) participation by women has increased strongly [6]. In addition, a Swiss study [13] showed that participants had a higher income level than non-participants. In that study, no differences in gender and educational level were found between participants and non-participants [13].

Next to identifying the socio-demographic characteristics of non-participants it is necessary to gain insight into other aspects as well. Socio-ecological models of health behavior posit that socio-demographic, psychological, social and environmental characteristics are all important determinants of health behaviors [14], and can impact participation in sport events as well [15]. An important premise of these socio-ecological models is the embedment of an individual within his or her social and physical environment [14]. Consequently, it is necessary to similarly focus on different levels of determinants (e.g., psychological and social environmental) in research. Furthermore, psychological aspects like perceived benefits, perceived barriers and motivation, and social aspects like social support of important others can be framed within the Theory of Planned Behavior [16] and the Self-Determination Theory [17]. The Theory of Planned Behavior [16] states that health behavior is determined by intention, that is in its turn determined by attitude (composed of perceived benefits and barriers), behavioral control (linked with perceived barriers) and subjective norms. Additionally, views and opinions of important others (e.g., social support) impacts these three factors. The Self-Determination Theory [17] states that the quality of motivation (autonomous versus controlled) is an important determinant of human behavior, including physical activity and sports. 

However, very little is known about these broader psychological and social aspects related to non-participation in running events. The few available studies suggest that individuals with a higher level of involvement in sports/physical activity participate more regularly in running events [18] and that motivational aspects play an important role in determining participation in sport events [19]. Furthermore, physically active individuals are more likely to participate in running events than inactive individuals [8]. It is known that motivating inactive individuals to participate in mass running events is very challenging, but a previous study showed that it was possible to motivate inactive adults to participate in accessible, short-distance park runs [10]. Nonetheless, evidence is lacking and other factors from the different layers of the socio-ecological framework (e.g., social support, perceived benefits, perceived barriers, self-efficacy, accessibility, convenience) that have consistently been associated with overall physical activity in adults [12] have rarely been examined with regard to participation in running events. Consequently, most aspects of the profile of non-participants remain to be identified.

Specifically for running events, the perception of event-specific barriers may play a prominent role in determining non-participation: obtaining insight in these barriers is necessary to find out why individuals are not participating. Previous research in participants of running events showed that the price-quality balance of the event, location, atmosphere and the attractiveness of the track of the running event are the most important predictors of future participation [20,21]. To the best of the authors’ knowledge no studies have examined the importance of event-specific factors/barriers to determine participation in individuals who do not participate in running events. Nonetheless, in order to tailor promotional actions towards people who are currently not participating in running events, it is necessary to gain insight in the specific barriers preventing them from participating. 

Based on the gaps in the current literature, the first aim of this pilot study was to examine which socio-ecological factors (i.e., socio-demographic, psychosocial and activity-related factors) are related to participation in running events. The current study focused on short- and middle-distance running events and not on marathons, since participating in a marathon requires very intensive training that is often so physically demanding and requires such a high degree of fitness that it eliminates a large degree of the population from ever participating. The second aim was to give an overview of the event-specific barriers for participation. Furthermore, building on the results of the first aim, a sub-aim was to investigate which barriers are mainly present in those adults having characteristics associated with a lower odds of participating in running events (i.e., those adults who are least likely to participate in running events and are thus most in need for effective promotional strategies). As a third aim, the current study examined differences in barriers towards participation in running events depending on gender, age and educational level. Results of this study can help to develop large-scale studies in this research domain and to tailor promotional actions for such events towards specific population subgroups in order to reach as many people as possible. 

## 2. Materials and Methods 

### 2.1. Procedure and Participants

Flemish adults (18–75 years) were asked to complete an online questionnaire, developed through the freely available software LimeSurvey (https://www.limesurvey.org/en/). Recruitment took place through purposeful convenience sampling using email and social media. The research team sent invitation emails to their professional and non-professional acquaintances and shared the link to the questionnaire through social media (Facebook, Twitter). Additional participants were recruited through snowball sampling (acquaintances were asked to share the link through email/social media).

The online questionnaire was available from the beginning of December 2015 until the end of March 2016 and it took on average about 15 minutes to answer all questions. In the invitation emails and when sharing the link, the study was introduced briefly and it was emphasized that participants as well as non-participants in running events were targeted. In the survey the following information was assessed: sociodemographic characteristics, psychosocial factors regarding physical activity, overall physical activity levels, participation in running events and experienced barriers towards participation in running events. 

In total, 313 participants completed the questionnaire. Because four of these participants were younger than 18 years and one participant had a chronic condition that prevented him/her from being active, the final analytical sample consisted of 308 adults. Informed consent was automatically obtained when the participants voluntarily completed the survey. The study was conducted in accordance with the Declaration of Helsinki, and the protocol was approved by the Ethics Committee of the Ghent University Hospital (EC/2015/1361). 

### 2.2. Measures

#### 2.2.1. Socio-Demographic Characteristics

The following socio-demographic characteristics were assessed: gender, age, educational level (primary school, secondary school, high school/college, university) and living environment (urban, suburban, rural).

#### 2.2.2. Psychosocial Factors

Four categories of psychosocial factors were included in the questionnaire: motivation towards physical activity, perceived benefits of physical activity, social support to participate in physical activity and knowledge about running events. 

Motivation towards physical activity was assessed using the Dutch version of the Behavioral Regulations in Exercise Questionnaire (BREQ-II) [22]. This questionnaire was developed to measure motivation towards exercise and showed to be sufficiently valid for use in adults [22]. Because exercise is only one part of overall physical activity, we replaced “exercise” by “physical activity” in the questionnaire. The questionnaire comprises 19 items relating to five motivation types from the self-determination theory [17], namely amotivation, external regulation, introjected regulation, identified regulation and intrinsic motivation. Each item is assessed on a five-point Likert-scale ranging from strongly disagree to strongly agree. The mean of the five subscales is calculated to have an estimation of the extent of motivation in each motivation type separately. For this study, the Relative Autonomy Index (RAI) was calculated. The RAI can be used to gain insight in the degree of relative autonomy, given that the five motivation types are located on the self-determination continuum. The RAI is calculated by using the following weighted formula: (amotivation * −3) + (external regulation * −2) + (introjected regulation * −1) + (identified regulation * 2) + (intrinsic regulation * 3). The minimum score for the RAI is −24 and the maximum score is +20. Higher positive scores for the RAI indicate more autonomous motivation whereas lower negative scores indicate less autonomous motivation. 

Perceived benefits of physical activity and social support from family and friends to participate in physical activity were assessed using questions derived from previous studies in adults and adolescents [23,24]. For perceived benefits of physical activity, two scales were constructed based on an exploratory factor analysis: physical benefits (e.g., better health; mean of three items; Cronbach’s alpha [α] = 0.54) and social benefits (e.g., meeting new people; mean of three items; α = 0.75). For social support, two scales were constructed: social support from family (e.g., encouragement to be active; mean of three items; α = 0.79) and social support from friends (e.g., being active together with friends; mean of three items; α = 0.84). All items were scored on a five-point Likert scale ranging from strongly disagree to strongly agree (perceived benefits) and from never to very often (social support). 

Knowledge about running events was assessed by presenting the participants a list of 30 running events. The list consisted of the most popular city runs (*n* = 17), obstacle runs (*n* = 9) and trail runs (*n* = 4) organized yearly in Flanders. Distances of the events ranged between two and 25 kilometers and most of the events offered more than one distance (e.g., “Straight across Bruges” = city run offering distances of 5 km and 15 km). Participants were asked whether or not they knew these events. Also the option “other” was provided to give the participants the opportunity to report events that were not included in the list. Scores for “knowledge” could range between 0 and 31 (i.e., knowing all events from the list and other events not in the list). 

#### 2.2.3. Physical Activity

Physical activity was assessed using the International Physical Activity Questionnaire (IPAQ), short usual week version (available at www.ipaq.ki.se). Reliability of the short IPAQ was found to be acceptable with a pooled ρ = 0.79 across 12 countries. Criterion validity assessed against accelerometers was fair to moderate with a pooled ρ = 0.30 [25]. Frequency (days/week) and duration (minutes/day) of walking, moderate physical activity and vigorous physical activity were assessed. For the current analyses min/week (i.e., days * min/day) of walking was calculated, as well as min/week of moderate-to-vigorous physical activity (sum of min/week of moderate and min/week of vigorous physical activity).

#### 2.2.4. Participation in Running Events

Participation in running events was assessed by one question, additional to the question on knowledge about running events (see above): “In how many of the running events you know did you participate during the past year?” Distance of the event one participated in was not taken into account. For instance, if respondents participated in “Straight across Bruges”, they were classified as “participating” independent of whether they participated in the 5 km or 15 km run. For the analyses this variable was dichotomized into no participation versus participation in at least one running event. 

#### 2.2.5. Barriers towards Participation in Running Events

Participants were asked about potential barriers preventing participation, except for those individuals that did participate in all events listed (*n* = 9). A list of 10 potential barriers was compiled during an expert meeting with two behavioral research scientists and two psychologists, and was based on previous research on perceived barriers toward physical activity [23,24,26]. The following barriers were queried: lack of interest, lack of time, lack company/encouragement, disappointment after a previous event, financial barriers, bad physical condition, distance to the event, insufficiently challenging, annoyance because of spectators and too much similarities compared to other running events. All items were assessed on a five-point Likert scale ranging from never to very often. 

### 2.3. Data Analyses

All analyses were conducted in SPSS 23.0 (IBM Belgium/Luxembourg, Brussels, Belgium) (SPSS data file Table S1). To examine the socio-demographic, psychosocial and activity-related correlates of participation in running events, a binary logistic regression analysis was conducted. Participation in a running event during the past year (yes/no) was included in the model as the dependent variable; three socio-demographic factors (= gender, age, educational level), six psychosocial variables (= RAI, physical benefits, social benefits, knowledge, social support from family, social support from friends) and two activity-related variables (= min/week walking, min/week moderate-to-vigorous physical activity) were included as independent variables. Descriptive statistics were used to describe the barriers towards participation present in the overall sample and in those adults with characteristics related to a lower odds of participation in running events (i.e., results of the binary logistic regression analysis). To examine the differences in barriers towards participation depending on gender (men versus women), age (<24 years, 24–40 years, >40 years) and educational level (high versus low), three one-way MANOVA analyses were conducted. Statistical significance was set at *p* < 0.05 for all analyses. 

## 3. Results

### 3.1. Descriptive Characteristics of the Sample

Socio-demographic, psychosocial and activity-related characteristics of the study sample are presented in Table 1. Overall, 66.9% of the sample was female, 79.5% had a college or university degree, 37.0% lived in an urban environment and 9.1% in a rural environment. Mean age was 31.8 years (standard deviation = 11.8). In total, 203 individuals did not participate in a running event during the past year and 105 individuals participated in at least one running event. Of these participants, 38 participated in one event, 24 in two events, 14 in three events, 17 in four or five events and 12 individuals participated in more than five events (with a maximum of 10).

### 3.2. Socio-Demographic, Psychosocial and Activity-Related Correlates of Participation in Running Events

Results of the binary logistic regression analysis are shown in Table 2. The analysis revealed that the RAI, social support from family, knowledge about events and min/week of moderate-to-vigorous physical activity were significantly associated with participation in running events. Adults with more autonomous motivation (OR = 1.142; 95% CI = 1.049, 1.243), perceiving more social support from family (OR = 1.509; 95% CI = 1.073, 2.122), having more knowledge about events (OR = 1.319; 95% CI = 1.215, 1.432) and with more min/week of moderate-to-vigorous physical activity (OR = 1.002; 95% CI = 1.000, 1.003) were more likely to have participated in at least one running event during the last year than adults with less autonomous motivation, less social support from family, less knowledge about events and lower levels of moderate-to-vigorous physical activity. For the socio-demographic and the other psychosocial and activity-related factors, no significant results were found.

### 3.3. Ranking of Perceived Barriers towards Participation in the Total Sample and in Adults with Characteristics Associated with Lower Odds of Participating in Running Events

In the total sample (*n* = 299; nine of the 308 respondents were excluded because they had participated in all running events they knew of), the top three of perceived barriers towards participation consisted to lack of time, bad physical condition and lack of interest. Financial barriers and lack of company/encouragement completed the top five (see Table 3). In adults who participated in at least one running event during the last year, the top three consisted of lack of time, distance to the event and financial barriers. In non-participants, having a bad physical condition, lack of interest and lack of time were the three main barriers towards participation. 

Based on the results of the binary logistic regression analysis (study aim 1), participants scoring “low” on the psychosocial (RAI, social support from family and knowledge about events) and activity-related (min/week of moderate-to-vigorous physical activity) factors associated with participation in running events were selected. This was done using a median split (i.e., selection of participants scoring lower than the median score) for the RAI, social support from family and knowledge about events. For min/week of moderate-to-vigorous physical activity, those who did not reach the health guideline for adults of 30 min moderate-to-vigorous physical activity per day were selected. Table 3 shows a ranking based on the importance (i.e., average item scores) of each potential barrier preventing participation in the different subgroups. In all subgroups, the top three consisted of the same barriers and was equal to the top three of the total sample: bad physical condition, lack of time and lack of interest. Furthermore, just like in the total sample, lack of company/encouragement and financial barriers completed the top five, except in adults who did not reach the health guidelines for physical activity: those adults perceived “annoyance because of spectators” as more important than financial barriers.

### 3.4. Differences in Barriers towards Participation Depending on Gender, Age and Educational Level

Descriptive statistics of the barriers towards participation depending on gender, age and educational level are presented in Table 4. One-way MANOVA analyses revealed differences in perceived barriers between men and women (multivariate F = 4.30, *p* < 0.001) and between the different age groups (multivariate F = 3.65, *p* < 0.001). Regarding gender, univariate analyses showed that having a bad physical condition was rather perceived as a barrier in women than in men (F = 21.54, *p* < 0.001), while perceiving an insufficient level of challenge was more prevalent in men than in women (F = 13.88, *p* < 0.001). Regarding age, univariate differences between age groups were found for lack of time (F = 10,83, *p* < 0.001), lack of company or encouragement (F = 5.80, *p* = 0.003), financial barriers (F = 9.31, *p* < 0.001) and distance to the event (F = 11.18, *p* < 0.001). 

Post-hoc analyses showed that lack of time was rather perceived as a barrier in the <24 year (*p* = 0.008) and the 24–40 year old participants (*p* < 0.001) than in the older (>40 years) participants. Financial barriers and a large distance to the event were more important barriers for the young (<24 years) participants than for the 24–40 year old individuals (*p* = 0.010 and *p* = 0.025 respectively) and those older than 40 (both *p* < 0.001). Finally, lack of company or encouragement was more prevalent in the <24 year old adults than in those older than 40 (*p* = 0.004). For educational level, the multivariate model was non-significant (F = 1.004, *p* = 0.440). However, univariate analyses showed that the barrier “annoyance because of the presence of spectators” was more prevalent in higher-educated adults than in lower-educated adults (F = 4.07, *p* = 0.044).

## 4. Discussion

The first aim of this study was to examine which socio-ecological factors (i.e., socio-demographic, psychosocial and activity-related factors) are related to participation in running events. Our findings showed that individuals who were more autonomously motivated towards physical activity, perceived more social support from their family, were aware of more running events and had higher levels of moderate-to-vigorous physical activity, were more likely to have participated in at least one running event during the last year. Furthermore, socio-demographic factors (gender, age and educational level), perceived social and physical benefits of physical activity, social support towards physical activity from friends and min/week of walking were not associated with participation in running events.

Our results confirm the democratization among participants in running events [27] as no differences in participation were found according to gender, age and educational level. This is a positive trend and confirms the hypothesis that running events have the potential to stimulate activity in population subgroups that are known to be less active (women and lower-educated adults) [12]. Nonetheless, this finding should be interpreted with caution because our sample mainly consisted of higher-educated individuals (79.5%).

Next to these socio-demographic factors, also motivation, perceived benefits, barriers, knowledge and social support were examined as potential correlates of participation in running events. These factors can be framed within the Theory of Planned Behavior [16] and the Self-Determination Theory [17]. Several psychosocial factors (i.e., motivation towards physical activity, social support from family, knowledge) that have consistently been associated with physical activity in adults [12,28] were also associated with participation in running events. A previous study already showed that motivational aspects can play an important role in determining participation in sport events [19]. This is in line with the self-determination theory [17], stating that the quality of motivation (autonomous versus controlled) is an important determinant of human behavior. A systematic review of Teixeira and colleagues [29] also found consistent proof that more autonomous forms of motivation (i.e., intrinsic motivation, identified regulation, integrated regulation, or a combination) are positively associated with physical activity and/or sports. As stated by the self-determination theory, strategies to increase autonomous forms of motivation should focus on satisfying the three basic psychological needs, namely autonomy, competence and relatedness [30]. Personal coaches (in real life or virtual through mobile applications) can play an important role regarding the fulfillment of these needs: by applying a motivating coaching style, for instance by providing positive feedback and stimulating feelings of connectedness and friendship [31], autonomous forms motivation can be stimulated: this may increase overall physical activity and indirectly affect participation in running events. 

No previous studies examined social support towards physical activity in relation to participation in running events. However, it is remarkable that in the present study, only social support from family and not from friends was related to participation in running events. No clear explanation for this finding can be given. Nonetheless, a recommendation for future studies could be to include specific questions about social support related to participation in running events instead of support related to overall physical activity; this may lead to different results and clarify the current findings. 

Next to motivation towards physical activity and social support from family, also knowledge about running events was related to participation in running events. Promotional campaigns (e.g., advertisement on television or in magazines) increasing adults’ knowledge of running events, may stimulate participation in such events. However, an important issue that needs to be taken into account when interpreting this finding, is the cross-sectional nature of our study. No conclusions about causality can be drawn and it is plausible that adults who already participate in running events have more knowledge because at running events there usually is extensive advertisement for other events. 

Finally, regarding the activity-related correlates, only moderate-to-vigorous physical activity was associated with a higher likelihood to participate in running events; the amount of walking/week was not related to participation. Previous studies also showed that mainly active adults participate in running events, although this may depend on the running distances offered in the event: shorter distances seem to attract less active participants [8]. Unfortunately our sample size was insufficiently large to conduct analyses stratified on distance of the running event. In this study a retrospective survey was used and “usual” levels of physical activity were examined in relation to participation in running events during the past year. Consequently, it is logical that those who have higher “usual” levels of physical activity, i.e., those who may have been training in preparation for a running event, are more likely to participate in a running event. For future research it would be more informative to use a longitudinal design, making it possible to link baseline physical activity levels (e.g., three months before a running event) with participation in running events at follow-up. An interesting finding was that walking was not associated with participation in running events, so a more intense dose of physical activity seems to be needed to stimulate participation in running events. Furthermore, it may be the case that people attracted to walking are not interested in running.

The second aim of this study was to give an overview of the event-specific barriers preventing participation in the overall study sample. Furthermore, building on the results of the first research aim, a sub-aim was to investigate which barriers are mainly present in those adults having characteristics associated with a lower odds of participating in running events (i.e., adults with low levels of autonomous motivation, knowledge, social support and moderate-to-vigorous physical activity). In the overall sample, the three main barriers were lack of time, lack of interest in running events, and having a bad physical condition. Furthermore, financial barriers and lack of company/encouragement completed the top five. When looking specifically at the main barriers in adults who are less likely to participate in running events, results were very similar. 

The main reported barriers were very similar to the barriers that have been previously associated with physical activity in adults [32,33]. Somewhat unexpectedly, the more general barriers (e.g., lack of time and lack of interest to participate in running events) were more important than the event-specific barriers (e.g., distance to the event, presence of spectators, level of challenge). Only in individuals who participated in at least one running event during the last year, other barriers were more prominent: next to lack of time, distance to the event and financial barriers completed the top three. Furthermore, lack of interest and having a bad physical condition were not part of the top five. These discrepancies confirm to need to conduct research in non-participants as well, as this seems to be a distinct group perceiving other barriers than adults who already participate in running events. Previous studies focused almost exclusively on participants and found that the atmosphere, distance to the event and attractiveness of the track were important factors determining re-participation [18,19]. Financial factors emerged as an important barrier in this study: participation fees for city runs in Flanders usually range between 10 and 30 EUR, while rates can run up to 50 EUR to participate in an obstacle run. Efforts to decrease these rates may lead to increased participation. 

Based on the current results, it seems that similar promotional strategies can be used across different types of non-participants (e.g., adults who are not autonomously motivated, adults who perceive few social support, inactive adults). Strategies that aim to recruit adults who are less likely to participate in running events, as well as overall strategies to recruit participants, should focus on overcoming these specific barriers. A potential approach could be by convincing individuals that the barriers can easily be tackled. For example, organizers of running events could try to overcome the barrier “lack of time” by specifically promoting their event to participants living nearby the event. By doing so, they can emphasize that it does not take much time to participate in an event near home. To tackle the barrier “lack of interest” organizers can highlight the health benefits of participating in running events, or emphasize the “fun” and cultural aspects of their event. Since a few years, several novel types of city runs are being organized in Flanders, such as runs of which the track passes through buildings one usually cannot enter freely (e.g., harbor buildings, museums), or color runs. Such innovative runs may have the capability to increase interest in people who perceive “lack of interest” as a main barrier. Additionally, it should be stressed that the training in preparation for an event can be easily integrated in daily life and does not necessarily take much time (e.g., by using start-to-run schemes). Furthermore, offering group subscriptions at a cheaper rate than individual subscriptions could be a strategy to tackle the financial barriers and encourage participation together with friends or family. Finally, event organizers could potentially overcome this barrier by providing training programs to prepare participants for that specific event or by equally promoting all different distances that are offered. The main and most promoted distance of an event often is a longer distance, while shorter distances are usually also offered at the same event. Potential participants should be motivated to participate in these shorter distances and should experience the feeling that participating in a shorter run is a big achievement.

As a final research aim, we examined if barriers towards participation in running events differed depending on gender, age and educational level. Results showed that the experienced barriers were relatively similar across sociodemographic subgroups, with some exceptions. For women, having a bad physical condition was more important while men rather perceived events as insufficiently challenging. Furthermore, lack of time, financial barriers, lack of company and a large distance to the event were mainly present in younger individuals while annoyance of spectators was more important in higher-educated adults than in lower-educated adults. 

Although the differences were small these distinct results according to age, gender and educational level should be taken into account when promoting participation in running events. Promotional campaigns could be tailored towards specific population subgroups, for instance by focusing on the level of challenge to recruit more men or by promoting group or student discounts to recruit younger participants. Previous research already emphasized that event organizers should be aware that their promotional strategy and the type of event they organize will attract specific participant profiles [15]. When organizers emphasize the competitive aspect of their event, mainly male and younger participants will be attracted to participate. Similarly, women and older adults will be attracted when the focus is put on health benefits and physical attractiveness and when shorter distances are offered [15].

A first strength of this study is the novelty of the research topic: the profile of participants in comparison with non-participants in running events was examined for the first time in Belgium, and this study provided very useful practical information for organizers of running events. Second, a relatively large sample was included in this study. Third, validated questionnaires were used to assess socio-ecological and activity-related factors. Furthermore, some important study limitations should be acknowledged. First, since our study sample was younger, more likely to be highly-educated and to be female than the Belgian population, generalizability of the results may be limited. Second, the use of an online questionnaire implies that specific segments of the population that are known to have less access to the Internet (e.g., older adults, lower-educated adults) are less likely to participate. This may have biased our results. Third, this study had a cross-sectional design making it impossible to draw any conclusions related to causality. Fourth, because of a lack of statistical power the analyses could not be stratified based on the distances offered in an event or the size of an event. In addition, many of the included events offered multiple distances, but no information was collected about which distance individuals participated in. Fifth, although this study was framed within theoretical frameworks like the socio-ecological framework [14], the Theory of Planned Behavior [16] and the Self-Determination Theory [17], it would be of added value to address this topic from the viewpoint of Choice Theories (e.g., Behavioral Choice Theory [34]). By doing so, perceived barriers and knowledge could be linked to the information-based choices individuals make regarding their participation in specific running events. Furthermore, in a broader context it would be interesting to examine whether participating in running events is listed on the “priority list” of individuals, and how this interacts with choosing alternative leisure activities. 

## 5. Conclusions

In conclusion, this study confirmed the democratization among participants of running events and showed that higher levels of autonomous motivation, social support from family, knowledge and moderate-to-vigorous physical activity were associated with a higher likelihood of participating in a running event. Furthermore, lack of interest, lack of time and having a bad physical condition were the three main barriers preventing adults from participation. Finally, with a few exceptions, perceived barriers were relatively similar across socio-demographic subgroups. Future longitudinal studies with a larger sample size are needed to confirm and refine the current results. 

## Figures and Tables

**Table 1:** Descriptive characteristics of the study sample.

Variable	Total Sample (*n* = 308)	Non-Participants ^a^ (*n* = 203)	Participants ^b^ (*n* = 105)
**Socio-demographic variables**			
*Gender (%)*			
Men	33.1	30.5	38.1
Women	66.9	69.5	61.9
*Age (mean [SD])*	31.3 (11.8)	31.1 (12.0)	31.8 (11.6)
*Educational level (%)*			
Primary school	0.3	0.5	0.0
Secondary school	20.1	19.7	21.0
High school/college	30.8	33.5	25.7
University	48.7	46.3	53.3
*Living environment (%)*			
Urban	37.0	34.0	42.9
Suburban	53.9	56.1	49.5
Rural	9.1	9.9	7.6
**Psychosocial variables**			
Relative Autonomy Index (mean [SD]) ^1^	8.4 (5.1)	7.2 (5.5)	10.8 (3.0)
Physical benefits (mean [SD]) ^2^	3.8 (0.7)	3.7 (0.7)	3.9 (0.6)
Social benefits (mean [SD]) ^2^	2.9 (1.0)	2.8 (1.0)	3.1 (1.0)
Social support family (mean [SD]) ^3^	2.3 (0.9)	2.2 (0.9)	2.5 (1.0)
Social support friends (mean [SD]) ^3^	2.9 (1.0)	2.8 (1.0)	3.2 (0.9)
Knowledge about events ^4^	9.3 (4.9)	7.5 (4.0)	12.9 (4.6)
**Physical activity**			
Min/week walking (mean [SD])	203.4 (258.4)	209.3 (263.1)	191.8 (250.0)
Min/week walking (median [IQR])	100.0 (195.0)	100.0 (185.0)	100.0 (230.0)
Min/week MVPA (mean [SD])	330.7 (247.1)	285.8 (240.0)	417.6 (238.5)
Min/week MVPA (median [IQR])	292.5 (330.0)	220.0 (320.0)	360.0 (300.0)

SD = standard deviation; IQR = interquartile range; MVPA = moderate-to-vigorous physical activity; **^a^** adults who did not participate in a running event during the last year; **^b^** adults who participated in at least one running event during the last year; **^1^** minimum -24, maximum 20; **^2^** five-point Likert scale from strongly disagree to strongly agree; **^3^** five-point Likert scale from never to very often; **^4^** minimum 0, maximum 31.

**Table 2:** Binary logistic regression analysis of socio-demographic, psychosocial and activity-related correlates of participation in running events.

Dependent variable: participation in running events: 0 = no participation during the last year, 1 = participation in at least one event during the last year			
Correlate	β (SE)	Odds Ratio	95% CI
Gender (ref: male)	0.006 (0.359)	1.006	0.498, 2.032
Age	0.017 (0.015)	1.017	0.988, 1.046
Educational level (ref: no college/univ)	0.126 (0.408)	1.135	0.510, 2.525
Relative Autonomy Index	0.133 (0.043)	1.142	1.049, 1.243 *
Physical benefits	0.249 (0.252)	1.282	0.783, 2.101
Social benefits	−0.106 (0.190)	0.899	0.620, 1.305
Social support family	0.412 (0.174)	1.509	1.073, 2.122 *
Social support friends	0.025 (0.200)	1.025	0.693, 1.516
Knowledge about events	0.277 (0.042)	1.319	1.215, 1.432 *
Min/week walking	0.000 (0.001)	1.000	0.999, 1.001
Min/week MVPA	0.002 (0.001)	1.002	1.000, 1.003 *

SE = standard error, 95% CI = 95% confidence interval, MVPA = moderate-to-vigorous physical activity; * *p* < 0.05

**Table 3:** Ranking of perceived barriers towards participation in the total sample and in adults who are less likely to have participated in a running event during the last year.

Barriers towards Participation ^1^	Total Sample (*n* = 299) Rank (Mean [SD])	Non-Participants (*n* = 203) Rank (Mean [SD])	Participants (*n* = 105) Rank (Mean [SD])	Low Relative Autonomy Index ^A^ (*n* = 155) Rank (Mean [SD])	Low Social Support from Family ^A^ (*n* = 150) Rank (Mean [SD])	Little Knowledge about Events ^A^ (*n* = 144) Rank (Mean [SD])	Not Reaching PA Guidelines ^A^ (*n* = 78) Rank (Mean [SD])
Lack of time	1 (3.36 [1.18])	3 (3.21 [1.17])	1 (3.64 [1.15])	2 (3.31 [1.19])	1 (3.26 [1.27])	3 (3.26 [1.21])	3 (3.20 [1.20])
Bad physical condition	2 (3.29 [1.40])	1 (3.65 [1.29])	6 (2.64 [1.36])	1 (3.78 [1.23])	2 (3.25 [1.43])	1 (3.64 [1.36])	1 (4.07 [1.15])
Lack of interest	3 (2.87 [1.36])	2 (3.29 [1.32])	8 (2.08 [1.05])	3 (3.03 [1.31])	3 (2.94 [1.40])	2 (3.37 [1.31])	2 (3.41 [1.35])
Financial barriers	4 (2.72 [1.38])	4 (2.57 [1.36])	3 (2.98 [1.39])	5 (2.66 [1.36])	4 (2.81 [1.43])	5 (2.47 [1.31])	6 (2.31 [1.34])
Lack of	5 (2.60 [1.21])	5 (2.57 [1.19])	5 (2.65 [1.25])	4 (2.65 [1.17])	5 (2.69 [1.25])	4 (2.77 [1.15])	4 (2.76 [1.19])
company/encouragement							
Distance to the event	6 (2.54 [1.19])	7 (2.27 [1.07])	2 (3.04 [1.26])	6 (2.47 [1.15])	6 (2.48 [1.21])	6 (2.31 [1.07])	7 (2.11 [1.11])
Annoyance spectators	7 (2.02 [1.18])	6 (2.31 [1.28])	10 (1.49 [0.71])	7 (2.36 [1.26])	7 (2.01 [1.18])	7 (2.26 [1.21])	5 (2.49 [1.36])
Similarities with other events	8 (1.99 [1.16])	9 (1.52 [0.85])	4 (2.87 [1.14])	8 (1.84 [1.03])	8 (1.99 [1.18])	9 (1.66 [0.92])	8 (1.62 [1.04])
Insufficiently challenging	9 (1.84 [0.97])	8 (1.64 [0.93])	7 (2.21 [0.95])	9 (1.79 [0.96])	9 (1.88 [1.02])	8 (1.70 [0.90])	9 (1.61 [0.96])
Disappointment	10 (1.42 [0.74])	10 (1.29 [0.65])	9 (1.66 [0.84])	10 (1.43 [0.75])	10 (1.50 [0.83])	10 (1.34 [0.62])	10 (1.30 [0.66])

MVPA = moderate-to-vigorous physical activity; SD = standard deviation; **^1^** all barriers were scored on a five-point Likert scale from never to very often; **^A^** median split was used to define. Groups scoring “low” on the respective psychosocial and activity-related variables.

**Table 4:** Perceived barriers towards participation in a running event: descriptive statistics and differences between socio-demographic subgroups (gender, age, educational level).

Barriers towards Participation Mean (SD) ^1^	Total Sample	Gender		Age			Educational Level	
		Men *n* = 99	Women *n* = 200	<24 *n* = 120	24–40 *n* = 118	>40 *n* = 61	No College/Univ. *n* = 60	College/Univ. *n* = 239
lack of interest	2.87 (1.36)	2.91 (1.40)	2.85 (1.35)	2.81 (1.29)	2.75 (1.40)	3.20 (1.41)	2.57 (1.17)	2.94 (1.40)
lack of time	3.36 (1.18)	3.39 (1.17)	3.35 (1.19)	**3.37 (1.16) ^A^**	**3.64 (1.11) ^B^**	**2.80 (1.18) ^A,B^**	3.18 (1.14)	3.41 (1.19)
lack of company/encouragement	2.60 (1.21)	2.59 (1.23)	2.61 (1.20)	**2.84 (1.18) ^A^**	**2.55 (1.22)**	**2.21 (1.14) ^A^**	2.53 (1.11)	2.62 (1.23)
disappointment	1.42 (0.74)	1.43 (0.82)	1.41 (0.70)	1.41 (0.77)	1.43 (0.73)	1.41 (0.72)	1.35 (0.63)	1.44 (0.77)
financial barriers	2.72 (1.38)	2.59 (1.33)	2.78 (1.41)	**3.10 (1.35) ^A,B^**	**2.57 (1.36) ^A^**	**2.25 (1.32) ^B^**	2.70 (1.36)	2.72 (1.39)
bad physical condition	3.29 (1.40)	**2.78 (1.49)**	**3.55 (1.28)**	3.23 (1.35)	3.24 (1.38)	3.54 (1.52)	3.18 (1.48)	3.32 (1.38)
distance to the event	2.54 (1.19)	2.48 (1.19)	2.57 (1.20)	**2.88 (1.14) ^A,B^**	**2.47 (1.21) ^A^**	**2.03 (1.06) ^B^**	2.65 (1.18)	2.51 (1.20)
insufficiently challenging	1.84 (0.97)	**2.13 (1.12)**	**1.70 (0.86)**	1.85 (0.99)	1.83 (0.99)	1.84 (0.92)	1.85 (0.94)	1.84 (0.98)
annoyance spectators	2.02 (1.18)	1.86 (1.11)	2.11 (1.21)	2.18 (1.15)	1.85 (1.15)	2.07 (1.26)	**1.75 (0.91)**	**2.09 (1.23)**
similarities with other events	1.99 (1.16)	1.96 (1.12)	2.01 (1.18)	1.97 (1.14)	2.05 (1.22)	1.92 (1.10)	2.03 (1.18)	1.98 (1.16)

SD = standard deviation; **^1^** all barriers were scored on a five-point Likert scale from never to very often; **Bold** results represent significant differences between groups (gender, age or educational level); post-hoc tests for age group: same superscript characters (A, B) = significant difference between groups.

## Supplementary Materials

The following are available online at www.mdpi.com/1660-4601/14/11/1315/s1, Table S1: SPSS data file of the study.
